# Structure-activity studies of Mdm2/Mdm4-binding stapled peptides comprising non-natural amino acids

**DOI:** 10.1371/journal.pone.0189379

**Published:** 2017-12-11

**Authors:** Sharon Min Qi Chee, Jantana Wongsantichon, Jiawei Siau, Dawn Thean, Fernando Ferrer, Robert C. Robinson, David P. Lane, Christopher J. Brown, Farid J. Ghadessy

**Affiliations:** 1 p53Lab, Agency for Science Technology and Research (A*STAR), Singapore, Singapore; 2 Institute of Molecular and Cellular Biology, A*STAR, Singapore, Singapore; Aberystwyth University, UNITED KINGDOM

## Abstract

As primary p53 antagonists, Mdm2 and the closely related Mdm4 are relevant cancer therapeutic targets. We have previously described a series of cell-permeable stapled peptides that bind to Mdm2 with high affinity, resulting in activation of the p53 tumour suppressor. Within this series, highest affinity was obtained by modification of an obligate tryptophan residue to the non-natural L-6-chlorotryptophan. To understand the structural basis for improved affinity we have solved the crystal structure of this stapled peptide (M011) bound to Mdm2 (residues 6–125) at 1.66 Å resolution. Surprisingly, near identity to the structure of a related peptide (M06) without the 6-chloro modification is observed. Further analysis of linear and stapled peptides comprising 6-Me-tryptophan provides mechanistic insight into dual Mdm2/Mdm4 antagonism and confirms L98 of Mdm4 as a mutable steric gate. The results also highlight a possible role of the flexible hinge region in determining Mdm2/Mdm4 plasticity.

## Introduction

Advances in genetic engineering and chemical biology have enabled tractable incorporation of non-natural, modified amino acids into designer peptides and proteins [1, 2]. Expansion of chemical diversity beyond nature’s repertoire can impart significant improvements in desired functionality and/or completely novel properties. Examples include enhancements in affinity and catalysis, novel biophysical properties and introduction of conjugation tags, all contingent on modified amino acids incorporated either rationally or through combinatorial selection [3–7].

We have previously described a series of peptide antagonists targeting Mdm2, a key regulator of p53 function [8–12]. In common with all reported high-affinity binders, these peptides incorporate a signature p53-derived interacting motif comprising F, W and L residues that respectively dock into discrete hydrophobic pockets in the N-terminal domain of Mdm2 [13]. This series exemplified significant enhancements in binding affinity, cellular uptake and activity arising from incorporation of a hydrocarbon tether and replacement of the tryptophan with the non-natural L-6-chlorotryptophan. The most bioactive stapled peptide (M011) showed ~7-fold increase in Mdm2 binding affinity and ~59-fold enhanced cellular activity over the parental peptide. Intriguingly, M011 showed relatively poor binding (~200 fold reduced) to Mdm4, the Mdm2 paralog that also binds to p53 and inhibits its function [14]. The Mdm2 and Mdm4 N-terminal domains that bind p53 share considerable structural homology [15], and there is significant interest in the clinical development of potent small molecule and peptidic dual inhibitors [16–18].

Towards reconciling this functional disparity, we have solved the structure of M011 bound to Mdm2 (residues 6–125). The structure highlights ready accommodation of the 6-chlorotryptophan chlorine atom by the hydrophobic pocket. Minimal structural perturbation is observed when compared to the isomorphous structure of a highly similar peptide (M06) lacking the 6-chloro group bound to the same Mdm2 construct [19]. Homology modeling and site directed mutagenesis provide further insight into the dynamic role of Mdm4 L98 as a steric gate to modulate binding of peptides with modified side chains.

## Materials and methods

### Peptide synthesis

The stapled peptides M012 and its linear precursor (M012-L) were synthesized by Mimotopes (Australia). All other peptides were synthesised in-house. The (*i*, *i* +7) hydrocarbon linkage was generated by placing the olefin-bearing unnatural amino acids (*S*)-2-(4′pentenyl) alanine and (*S*)-2-(7′-octenyl) alanine at positions 4 and 11, respectively. These were then fused via olefin metathesis using the Grubbs catalyst. Peptides were purified using HPLC to >90% purity. All peptides were amidated at their C-terminus and acetylated at their N-terminus. The linear precursors M011-L and M012-L comprise (*S*)-2-(4′pentenyl) alanine and (*S*)-2-(7′-octenyl) alanine but have not been cyclised.

### Protein expression and purification

Mdm2 (amino acids 6–125) was cloned as a GST-fusion protein using the pGEX-6P-1 GST expression vector (GE Healthcare). The mutant Mdm2-M62A (amino acids 6–125) was generated using QuickChange site directed mutagenesis protocol (Stratagene). This mutation promotes crystallisation with no significant impact on binding of peptide antagonists [19]. The constructs were then transformed into *Escherichia coli* BL21(DE3) pLysS (Invitrogen) competent cells. Cells were grown in LB medium at 37°C and induced at OD_600 nm_ of 0.6 with 0.5 mM IPTG at 16°C. After overnight induction, the cells were harvested by centrifugation, resuspended in binding buffer (50 mM Tris-HCl pH 8.0, 150 mM NaCl), and lysed by sonication. After centrifugation for 60 minutes at 19,000 × g at 4°C, the cell lysate was then applied to a 5 mL GSTrap FF column (GE Healthcare) pre-equilibrated in wash buffer (50 mM Tris-HCl pH 8.0, 150 mM NaCl, 1 mM DTT). The proteins were cleaved on-column by PreScission protease (GE Healthcare) overnight at 4°C and eluted off the column with wash buffer. The protein sample was then dialyzed into buffer A solution (20 mM Bis-Tris, pH 6.5, 1 mM DTT) using HiPrep 26/10 Desalting column, and loaded onto a cation-exchange Resource S 1 mL column (GE Healthcare) pre-equilibrated in buffer A. The column was then washed in 6 column volumes of buffer A and bound protein was eluted with a linear gradient in buffer comprising 1 M NaCl, 20 mM Bis-Tris pH 6.5, and 1 mM DTT over 30 column volumes. Protein purity as assessed by SDS-PAGE was ~95%, and the proteins were concentrated using Amicon-Ultra (3 kDa MWCO) concentrator (Millipore).

Mdm4 and Mdm4-L98V (amino acids 1–108) were cloned with C-terminal 6xHIS tags. The constructs were then transformed into *Escherichia coli* BL21(DE3) (Invitrogen) competent cells. Cells were grown in LB medium at 37°C and induced at OD_600 nm_ of 0.6 at 25°C with 0.25 mM IPTG and at 16°C with 1 mM IPTG for Mdm4-WT and Mdm4-L98V respectively. After overnight induction, the cells were harvested by centrifugation, resuspended in His-binding buffer (50 mM Bis-Tris pH 6.5, 1M NaCl, 20 mM imidazole, 0.5 mM DTT), and lysed by sonication. After centrifugation for 60 mins at 19,000 × g at 4°C, the cell lysate was then applied to a 1 mL HisTrap FF column (GE Healthcare) pre-equilibrated in His-binding buffer, washed and eluted off the column with His-elution buffer (50 mM Bis-Tris pH 6.5, 1M NaCl, 500 mM imidazole, 0.5 mM DTT) with a gradient elution of 35% elution buffer in 5 column volumes, followed by 35% to 100% elution buffer over 80 column volumes. Protein purity as assessed by SDS-PAGE was ~ 90%, and the fractions were pooled and buffer exchanged into 50 mM Bis-Tris pH 6.5, 350 mM NaCl, 1 mM DTT. The proteins were then concentrated using Amicon-Ultra (3 kDa MWCO) concentrator (Millipore).

### Crystallization

The lyophilized stapled peptide M011 was first dissolved in DMSO to make a 10 mM stock solution. The purified proteins were concentrated to approximately 3.5 mg/mL and then incubated with the stapled peptide at a 1:3 molar ratio of protein to peptide at 4°C overnight. The sample was clarified by centrifugation before crystallization trials at 16°C using the sitting drop vapour diffusion method. Crystals of Mdm2-M62A in complex with stapled M011 peptide were obtained by mixing the protein-peptide complex with the reservoir solution in a ratio of 1:1, with the reservoir solution containing 0.04 M Citric acid, 0.06 M Bis-Tris propane pH 6.4, and 20% PEG 3350.

### Data collection and structure determination

X-ray diffraction data was collected at the Diamond Light Source (DLS, UK). Data collection was carried out as a series of contiguous 0.1° oscillations for 360° rotations at a wavelength of 0.979 Å. The Xia2 program package was used to index, integrate and scale data. Molecular replacement using the human Mdm2 structure (PDB 1YCR) as a model in PHASER [20] was used to initiate structure determination. Restrained refinement was performed using REFMAC 5.0 [21] in the CCP4 suite of programs [22]. Model building was carried out in COOT [23]. Jligand [24]was used to define and generate the geometric restraints for the non natural amino acids ((*S*)-2-(4′pentenyl) alanine) and ((*R*)-2-(7’octenyl) alanine) (which form the hydrocarbon staple in M011), the covalent bond linking their respective side-chains together to form the macrocyclic linkage and for 6-chloro-trptophan. Validation of the final refined structures was carried out using MOLPROBITY [25]. All structural figures were generated with PyMOL (Delano Scientific LLC). Atomic coordinates of the crystal structure have been deposited into the Protein Data Bank (PDB access code 5XXK). Crystallographic data collection and refinement statistics are shown in Table 1. See S1 File for PDB summary report.

**Table 1:** Crystallographic data collection and refinement statistics. Highest resolution bin data stated in parentheses. RMSD values correspond to the root-mean-square deviations of bond lengths and angles of the final restrained and refined structure from experimentally determined ideal values [26].

Crystallographic Data Table	
**Resolution (Å)**	**25.6–1.66 (1.70–1.66)**
**Space Group**	**P2**_**1**_ **2**_**1**_ **2**
**Unit Cell Dimensions (Å)**	**a = 65.5, b = 106.4, c = 39.3**
**Temp (K)**	**100**
**Redundancy**	**6.4 (6.3)**
**Collected Reflections**	**210773 (15241)**
**Unique Reflections**	**32933 (2420)**
**R merge (%)**	**4.7 (68.5)**
**I/sigma**	**16.6 (2.3)**
**R factor (%)**	**18.75**
**R free (%)**	**20.88**
**RMS Bonds (Å)**	**0.0094**
**RMS Angles (°)**	**1.452**
**% Completeness**	**99.4 (99.0)**
**Wilson B-Factor (Å**^**2**^**)**	**24.3**
**Average Refined B-Factors (Å**^**2**^**)**	
**Chain A**	**32.4 (Mdm2)**
**Chain B**	**34.0 (Mdm2)**
**Chain C**	**24.8 (M011)**
**Chain D**	**26.2 (M011)**
**Waters**	**44.0**
**Number of Solvent Molecules**	**124**
**Ramachandran Data**	
**Outliers (%)**	**0.00**
**Favoured (%)**	**99.47**

### Fluorescence anisotropy

Fluorescence anisotropy assays were performed as previously described [8]. Titrations of purified Mdm2/Mdm4 proteins were incubated with 50 nM of carboxyfluorescein (FAM) labelled 12–1 peptide (FAM-RFMDYWEGL-NH2) to initially determine the dissociation constants for the peptide-protein interactions. Apparent K_d_s of peptides were next determined by competitive fluorescence anisotropy. Titrations of peptides were carried out at constant concentrations of Mdm2/Mdm2-M62A/Mdm4 (150 nM), Mdm4-L98V (250 nM) and labelled peptide (50 nM). Anisotropy measurements were carried out using the Envision Multilabel Reader (PerkinElmer). All experiments were carried out in PBS (2.7 mM KCl, 137 mM NaCl, 10 mM Na_2_HPO_4_ and 2 mM KH_2_PO_4_, pH 7.4), 4% DMSO and 0.1% Tween-20 buffer. All titrations were carried out in duplicate (3–6 repeats). Curve fitting was carried out using Prism (GraphPad).

### p53 reporter assay in T22 cells

Dulbecco’s Minimal Eagle Medium (DMEM) with 10% fetal bovine serum (FBS) and penicillin/streptomycin was used to maintain cells. T22 cells stably transfected with a p53 responsive β galactosidase reporter [27], were seeded into 96-well plates (8000 cells per well). 24 hours later, cells were treated with compounds/peptide for 18 hours in DMEM with 10% FBS. β galactosidase activity was measured using the FluoReporter LacZ/Galactosidase Quantitation kit (Invitrogen). Measurements were carried out on a Safire II multiplate reader (TECAN). All experiments were carried out in duplicate.

### LDH leakage assay

T22 cells were seeded into a 96-well plate at density of 5000 cells/mL and incubated overnight. Cells were treated with the peptides (in presence of 10% FBS) with a final 1% (v/v) DMSO concentration. 0.1% TritonX-100 provided by the CytoTox 96^®^ Non-Radioactive Cytotoxicity kit (Promega) was added as a maximum LDH release control together with a negative control of 1% (v/v) DMSO. The assay was performed according to the manufacturer’s instructions. Absorbance readings were detected using an Envision multiplate reader (Perkin-Elmer).

### Statistical analysis

Statistical analysis (unpaired t-test) was carried out using GraphPad software.

## Results and discussion

### Structural determination of M011 bound to Mdm2-M62A

**Fig 1:**
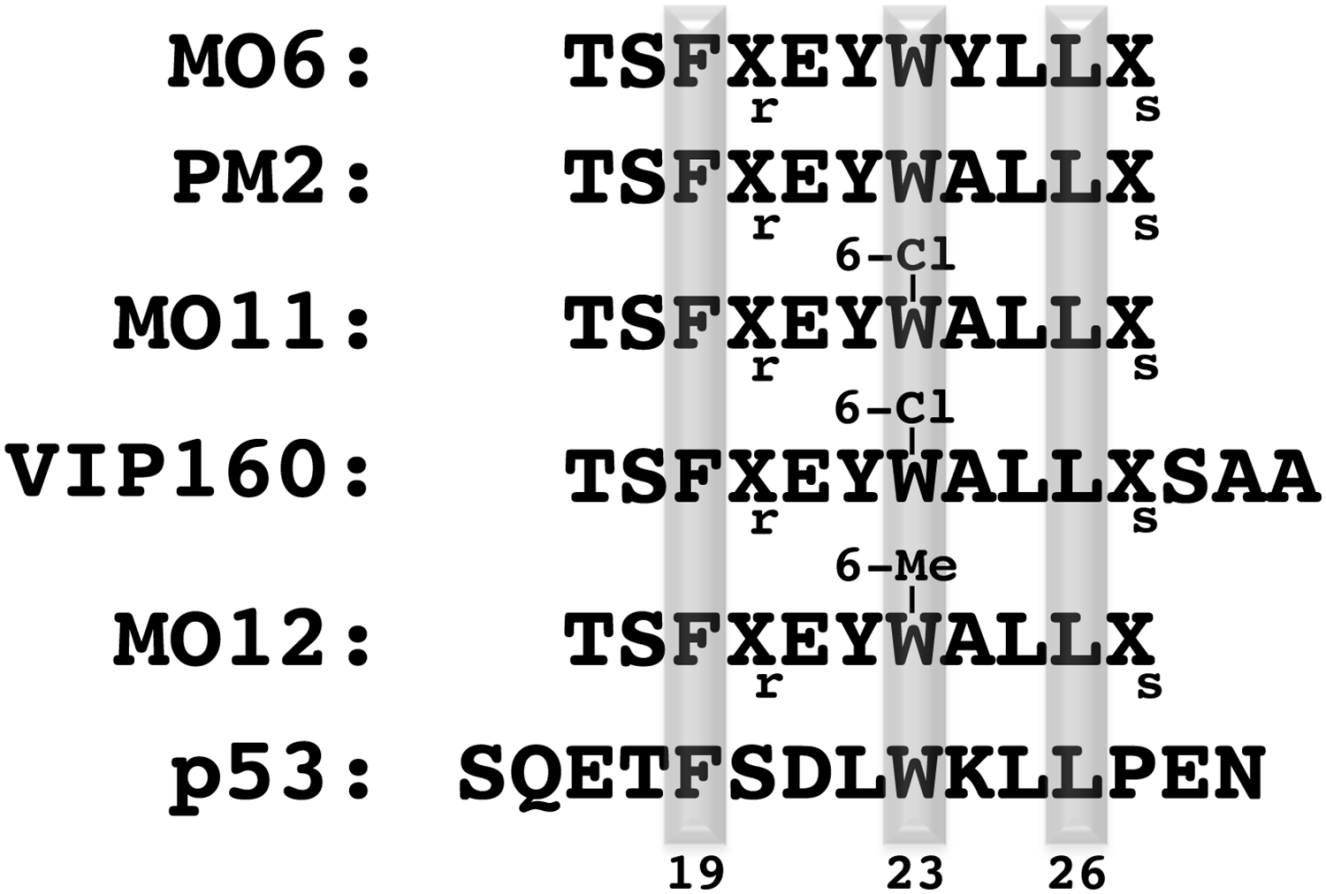
Sequence alignment of peptide ligands binding the Mdm2/Mdm4 N-terminal domains. Shaded residues are the conserved interacting residues (F19, W23 and L26) derived from p53. Xr and Xs represent (*S*)-2-(4′pentenyl) alanine and (*S*)-2-(7′-octenyl) alanine (coupled by olefin metathesis).

**Fig 2:**
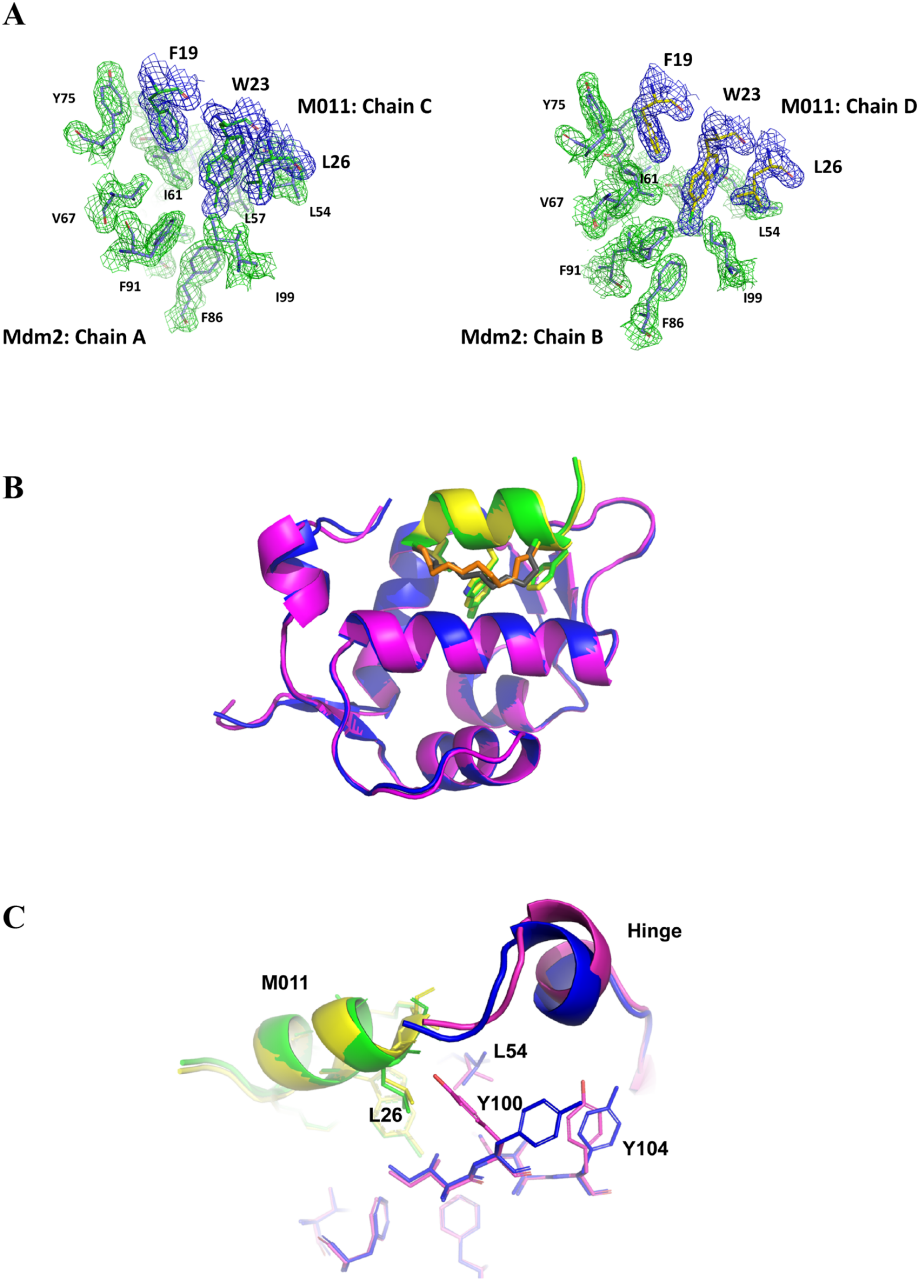
Crystallographic unique interaction sites of the M011 stapled peptide bound to the N-terminal p53 binding domain of Mdm2-M62A (6–125). A, The 2Fo-Fc electron density map, contoured at 1.5 σ, clearly denotes the amino acid residues involved in recognizing the conserved Mdm2 binding motif (F19, 6-Cl-W23 and L26, shown in bold). Green mesh highlights residues from Mdm2 and blue mesh indicates residues from the stapled M011 peptide. The residues involved in recognizing the M011 peptide in the other complex show negligible conformational differences. B, Overlay of the two unique complexes. The M011 peptide is highlighted in yellow with the staple colored orange (chain D) and green with the staple coloured charcoal (chain C), whilst Mdm2 (chain A) is shown in blue and Mdm2 (Chain B) is highlighted in magenta. For clarity only side chains of the interacting peptide residues (F19, 6-Cl-W23, L26) are depicted. C, Small conformational differences are observed in the hinge region of Mdm2 peptide binding pocket. Fluctuations in the lid/hinge region (residues 18–26) in conjunction with Y100 and Y104 induce a conformation change of the M011 L26 residue projecting into the Leu pocket of Mdm2. Colouring scheme as in B.

**Fig 3:**
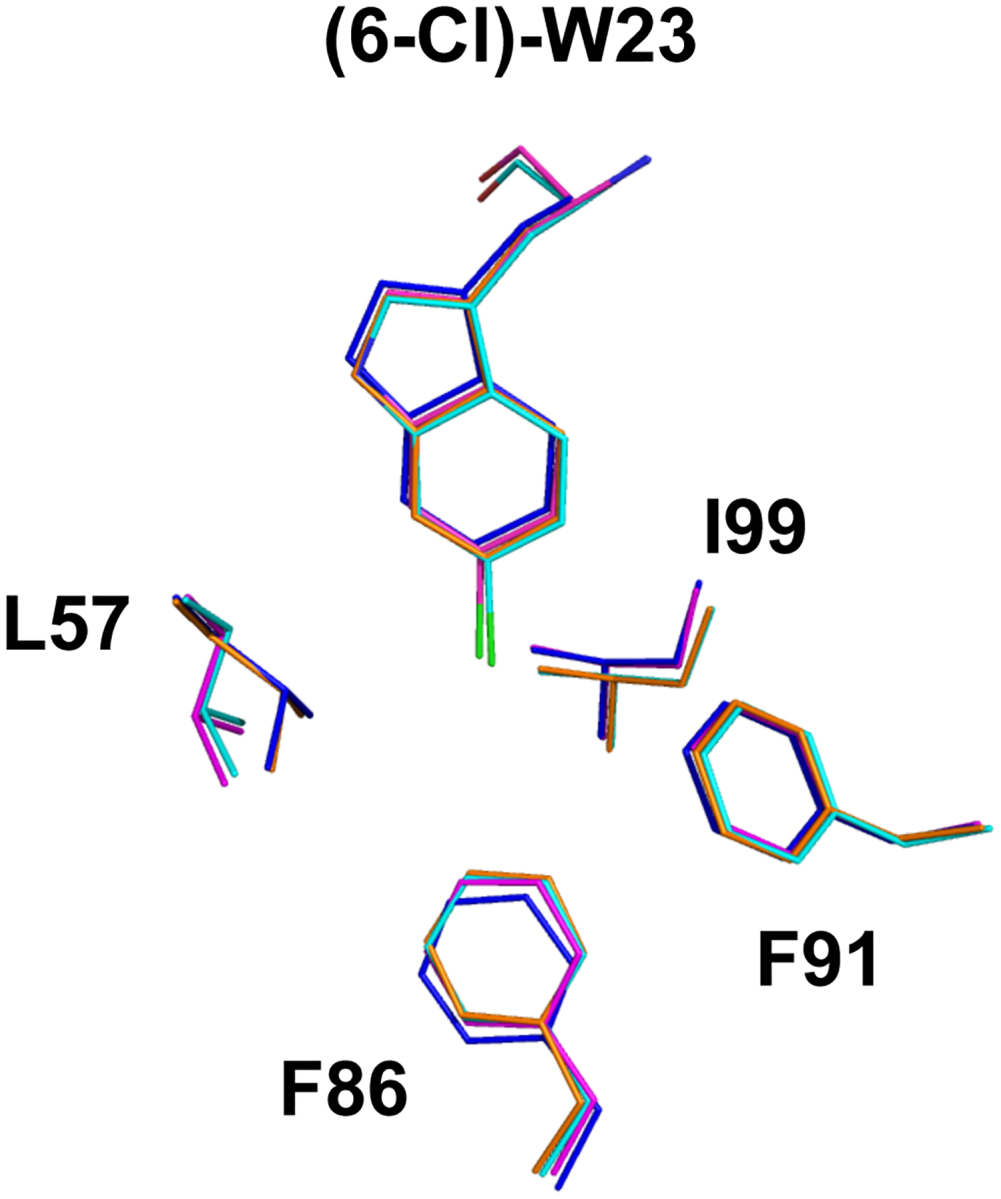
Structural overlay of M06 and M011 peptides bound to Mdm2. Only the tryptophan residue of each peptide (with 6-chloro group shown in green for M011) and amino acids forming the Mdm2 Trp pocket are depicted. M06: orange, blue; M011: cyan, magenta. Corresponding Mdm2 residues for each peptide are in the same colour. Image generated using structures 4UMN and 5XXK.

The sequence compositions of peptides used in this study are shown in Fig 1. The crystal structure of M011 bound to Mdm2-M62A shows 2 unique complexes in the asymmetric unit (Fig 2A, S1 Fig). Within each, a single molecule of M011 interacts with a pronounced hydrophobic cleft that binds endogenous p53 (residues 19 to 26). As with other peptide antagonists, an interfacing signature triad of F^19^, W^23^ (6-chloro) and L^26^ residues (numbering based on p53 sequence) project their side chains into discrete apolar sub-pockets (“Phe”, “Trp” and “Leu” pockets) (Fig 2B). Comparison with the structure of a similar stapled peptide (M06) [19], comprising native tryptophan bound to the same Mdm2 construct shows remarkable similarity. Each M011 complex, from the asymmetric unit, shows high structural similarity to the corresponding complex bound to M06 (all-atom derived RMSDs = 0.167 and 0.126 Å). This is particularly evident in the binding site, where minimal variation is observed in the ligand conformations (all-atom RMSDs = 0.055 and 0.031 Å). The major difference arises from rotation around the CHI1 bond of the L57 side chain in Mdm2 to widen the Trp pocket, with other residues comprising this pocket (F86, F91, I99) remaining unchanged (Fig 3). This optimal formation of Van der Waals contacts around the 6-chloro moiety, resulting from the change in conformation of L59, explains the ~8-fold increased binding affinity of M011 over the parental stapled peptide lacking the 6-chloro modification (PM2, Table 2). Structural differences are also observed in the lid/hinge region between the two M011: Mdm2 complexes in the asymmetric unit (Fig 2C). These result in a small conformational change of the M011 L26 residue. The observed changes in the hinge regions are primarily the result of crystal packing effects. However, these differences highlight how capping of the peptide binding groove by the hinge region can potentially impact on the binding of ligands to Mdm2.

**Table 2:** Apparent K_d_s (nM) for peptides binding to Mdm2/Mdm4 N-terminal domains and indicated variants. No significant variations are observed for binding of peptides to Mdm2-M62A (used in structural studies) compared to Mdm2. Values represent average ± SD (n = 2). Previously reported values are 34.35 ± 2.03 (_*_), 6.76 ± 2.11(_**_), 45.73 ± 7.65 (#) and 1360 ± 600 nM (##) [8].

	Mdm2	Mdm2-M62A	Mdm4	Mdm4-L98V
**PM2**	50.73 ± 12.27*	34.40 ± 7.90	100.89 ± 9.29^#^	72.32 ± 2.45
**M011**	6.61 ± 5.06**	6.337 ± 2.92	334.53 ± 45.17^##^	72.35 ± 7.91
**M011-L**	132.07 ± 55.79	39.79 ± 10.39	1388.07 ± 511.62	594.43 ± 40.51
**VIP160**	9.04 ± 1.11	11.05 ± 1.06	193.63 ± 9.15	76.12 ± 6.29
**M012**	17.847 ± 5.34	14.706 ± 7.24	1785 ± 310.14	385.03 ± 89.75
**M012-L**	13.21 ± 2.00	9.72 ± 2.52	410.8 ± 51.77	165.47 ± 26.52

### A stapled peptide comprising 6-Me-tryptophan shows in vitro and cell-based activity

The structural data suggests that bulkier substituents at the 6-position of the tryptophan indole ring should readily be accommodated, and could possibly contribute to increased affinity. We therefore generated the stapled peptide M012, replacing the chlorine in M011 with a methyl group. This modification has previously been shown to increase the affinity of linear peptides targeting Mdm2 [28]. Both M012 and a linear, pre-cyclised version (M012-L) bound Mdm2 efficiently as measured by fluorescence polarization assay (Table 2). Whilst their affinities did not significantly differ compared to M011, it is possible these values are underestimates. We could not prepare enantiomerically pure peptides due to unavailability of purified D- and L- 6-methyltryptophan isomers. In the case of M011, changing from the L- to D-isomer of 6-chloro tryptophan reduces affinity for Mdm2 by ~ 15 fold [8].

**Fig 4:**
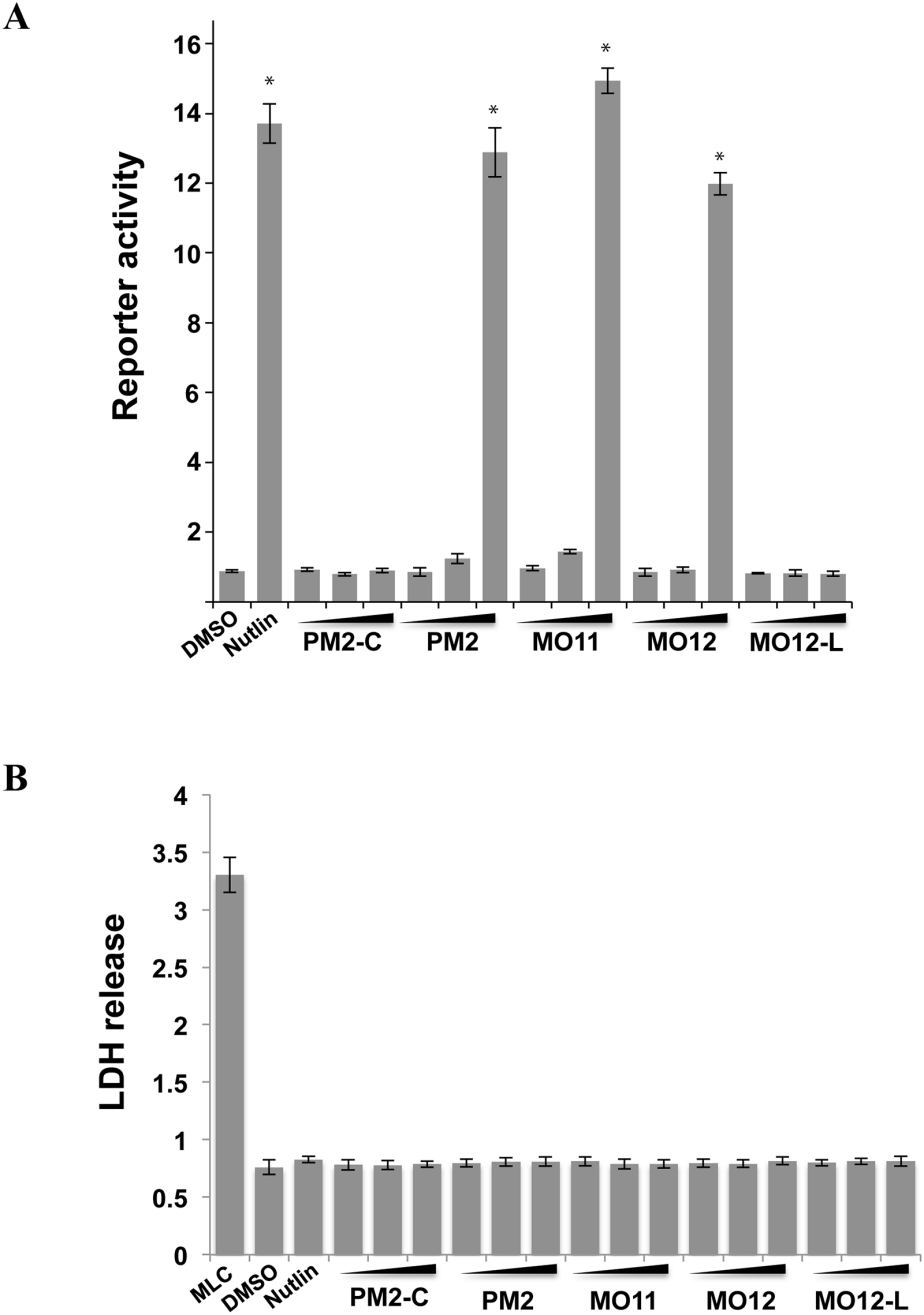
T22 reporter assay measuring p53 transactivation. A. Cells were treated with increasing concentrations (5, 10, 20 μM) of indicated peptide ligands and p53 activity determined by measuring β galactosidase levels. The small molecule Mdm2 inhibitor Nutlin (10 μM) was used as positive control. Activity is expressed as fold increase over cells treated with DMSO vehicle only. Values represent average ± SD (n = 2). * p < 0.001 compared to DMSO control. B. Lactate dehydrogenase release assay indicating no significant membrane disruption observed for increasing concentrations (2, 10, 20 μM) of indicated ligands compared to vehicle (DMSO) and positive maximum lysis control (MLC). Values represent average ± SD (n = 3).

M012 was next assayed in a cell-based assay to measure ensuing p53 activation upon inhibition of Mdm2 (Fig 4A). It showed comparable activity to the parental peptide with an unmodified tryptophan (PM2) and the small molecule antagonist Nutlin. M011 was ~20% more active than M012 in this assay. Pre-cyclised peptide (M012-L) showed no activity, highlighting the role of the staple in facilitating cellular uptake. Stapled peptides can sometimes damage cell membranes, with contextual hydrophobicity/charge cited as contributing factors [29]. Measurement of lactate dehydrogenase release by cells after peptide treatment showed no variation within the peptide series, indicating that the additional hydrophobicity of the 6-methyl group resulted in no significant changes in membrane viability (Fig 4B).

### L98 of Mdm4 is a mutable steric gate

**Fig 5:**
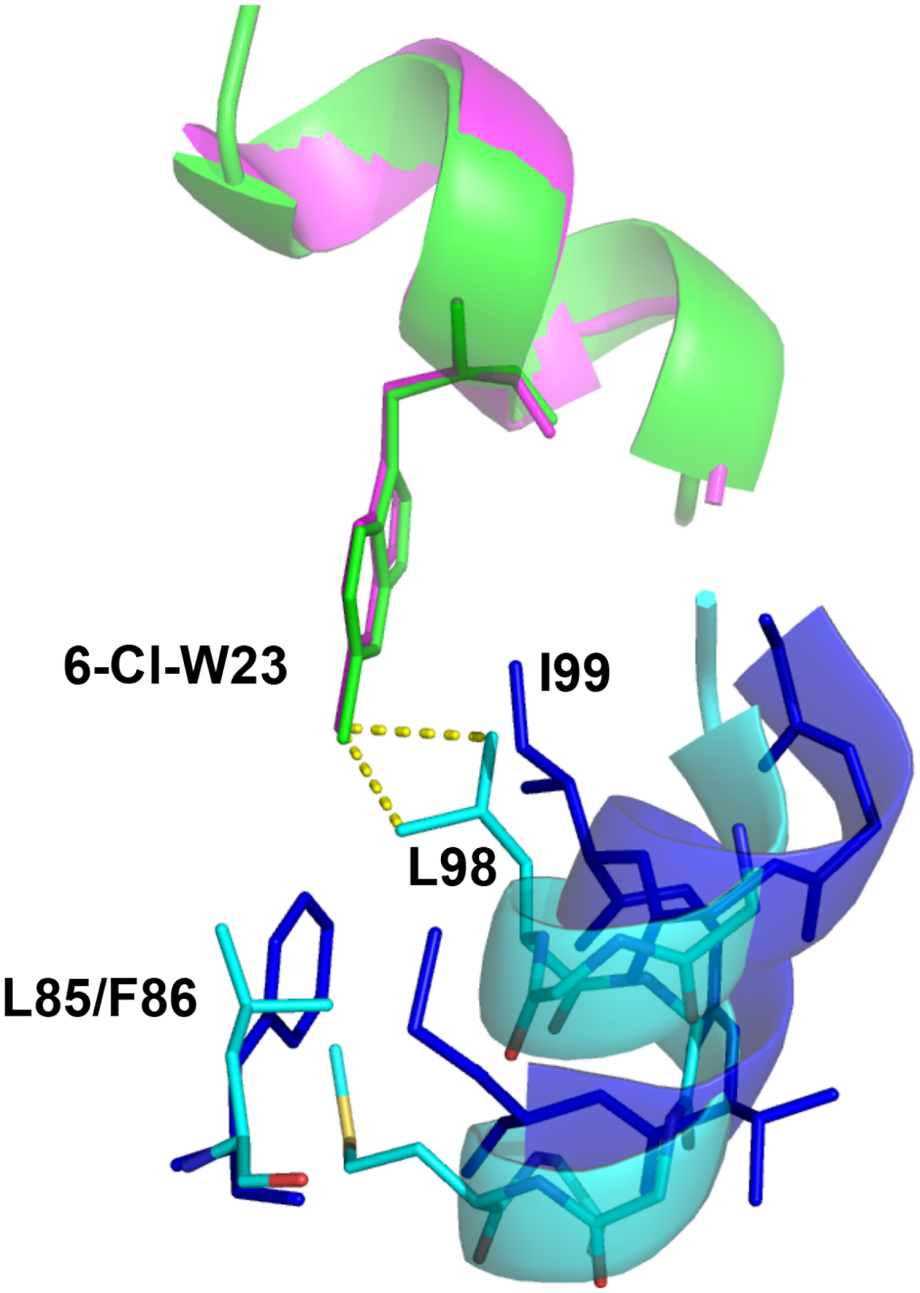
Overlay of residues comprising the Trp pocket in M011-Mdm2 and Cpd2-Mdm4 structures. M011 peptide (green) 6-chloro group of W23 projects deeper into the Mdm4 (cyan) Trp pocket compared to linear Cpd2 peptide (magenta), potentially clashing with the L98 side chain. The position of the corresponding I99 residue in Mdm2 (blue) results in a wider Trp pocket. The mutation of F86 in Mdm2 to L85 in Mdm4 results in the loss of a favorable Van der Waals interaction with the 6-chloro moiety of W23. In addition, the absence of F86 results in a conformational change in the α2 helix between the two proteins. This causes a reorientation of the L98 residue in Mdm4 in comparison to I99 of Mdm2 and brings it into closer proximity to the 6-chloro moiety, forming a less energetically favorable hydrophobic interaction. Several regions of Mdm2/Mdm4 have been omitted for clarity.

The p53-degrading function of Mdm2 is enhanced by the structurally related paralog Mdm4 (also known as MdmX) [30–33]. Whilst small molecule dual antagonists typically show biased affinities towards Mdm2 [18], peptides can efficiently target both Mdm2 and Mdm4 with near equivalency [34] [35]. Notably, whilst PM2 binds both Mdm2/Mdm4 efficiently, the 6-chloro modification of M011 results in ~3 fold reduced binding to Mdm4 compared to PM2. The affinity of M011 for Mdm4 is ~50 times reduced compared to Mdm2 binding affinity (Table 2) [8]. This bias was further compounded for M012, which showed ~100 fold increased binding to Mdm2 over Mdm4 (Table 2). Superimposition of the M011-Mdm2 structure onto Mdm4 highlights L98 of Mdm4 (equivalent to I99 of Mdm2) as a possible steric deterrent to accommodation of rigidified peptides with position 6-modifed indole rings (Fig 5). Indeed, the structure of a linear peptide with the 6-chlorotryptophan modification (cpd2) bound to Mdm4 shows crucial side chain reorientations of L98 and the absence of the F86 (equivalent to L85 in Mdm4) residue, found at the bottom of the pocket that the 6-chloro packs against [36]. In addition to removing favorable hydrophobic interactions, the mutation of F86 to L85 in Mdm4 also results in reorientation of the alpha-2 helix, causing the repositioning of L98 and other residues located at the N-terminal end of the helix. This residue was therefore mutated to valine to widen the base of the Trp pocket and alleviate the potential steric hindrance with the 6-chloro moiety on the peptide.

The binding affinities of both M011 and M012 to Mdm4-L98V showed ~4.6 fold increase over binding to Mdm4. Notably, binding of parental PM2 to Mdm4-L98V was only marginally improved (~ 1.4 fold), and both PM2 and M011 bound Mdm4-L98V with the same affinity (Table 2). The relatively high affinity of the linear cpd2 peptide for Mdm4 (36 nM measured by isothermal calorimetry) [36] suggested that the staple moiety itself might be an additional impediment to productive binding by M011 and M012. The inherent reduced flexibility of these peptides could be restricting reorientation of the modified W23 side chains to avoid steric conflicts with L98. In partial accord, the linear, pre-stapled version of M012 (M012-L) displayed ~4 fold higher affinity for Mdm4 over M012, with no change in affinity for Mdm2. However, pre-stapled M011 (M011-L) showed ~4 fold reduced binding to Mdm4 compared to M011 and also displayed ~20 fold reduced binding to Mdm2. Influence of the fully-formed staple moiety thus appears to be context dependent. It should be noted that in the absence of Trp^23^ modification, stapled peptides exhibiting very high affinity for Mdm4 (2.31 nM determined by surface plasmon resonance) have been described [37].

### Ordering of the Mmd2 hinge-region by M011 may influence pocket geometry

**Fig 6:**
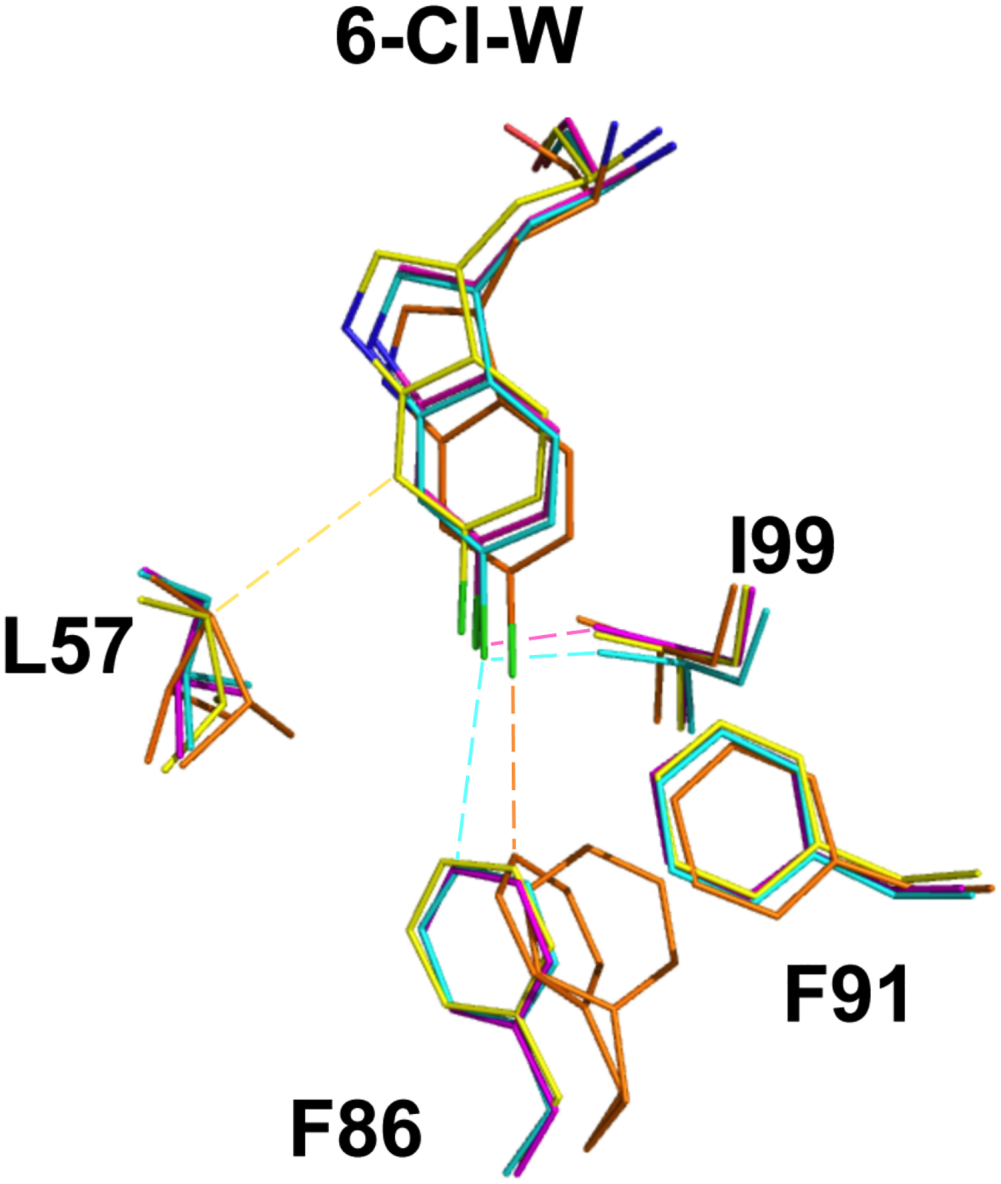
Structural overlay of M011, 78A, P2 peptides bound to Mdm2. Only the tryptophan residue of each peptide (with 6-chloro group shown in green) and amino acids forming the Mdm2 Trp pocket depicted. M011: cyan, magenta; 78A: orange; P2: yellow. Corresponding Mdm2 residues for each peptide are in the same colour. Image generated using structures 2GV2, 2AXI and 5XXK. Dotted lines represent the shortest Van der Waals contacts. Formulae for the complete P2 and 78A peptides are N-acetyl-Phe-Met—Phe(4-MePhos)-Trp(6-Cl)-Glu-(AC3C)-Leu amide and cyclo(-Phe-Glu-(6-Cl)Trp-Leu-Asp-Trp-Glu-Phe-d-Pro-Pro-), respectively.

The described structures of other peptides both with and without the 6-chloro tryptophan modification bound to Mdm2 reveal ligand-induced shifts in the conformations of the Trp and Leu pockets [13, 38, 39] (Fig 6). In particular, the structure of a macrocyclised β-hairpin peptide with the corresponding 6-chlorotryptophan (peptide 78A) shows significant variations in the side-chain geometries of residues forming the base of the Trp pocket (F86, F91, L57 and I99) [38]. However, this is mainly due to the hairpin alpha-helical mimetic maximizing its interaction surface area with Mdm2, resulting in deeper burial of the 6-chloro tryptophan and the conserved leucine projecting less deeply into the Leu pocket, allowing the F, L and W residue on the opposing strand of the hairpin to also pack efficiently against Mdm2.

In contrast, only limited structural variations within the Trp and adjacent Leu pockets are seen when comparing the M06 and M011 structures Here, ordering of the hinge region (residues 20–24) extends the periphery of the Leu pocket and effectively caps the hydrophobic cleft (Fig 2). This tight capping may reduce plasticity of both the Leu and the adjacent Trp pocket, accounting for the highly similar M06 and M011 structures. Similar ordering of the corresponding hinge region in Mdm4 could also inhibit remodeling of the Trp and Leu pockets required for efficient binding of bulkier ligands [36]. In this case, steric clashes that would otherwise inhibit productive binding can be resolved by side directed mutagenesis of Mdm4, as observed for improved binding of M011/12 to the L98V mutant.

The results indicate that binding of M011/12 stapled peptides to Mdm2/Mdm4 likely induce structural changes that suppress plasticity and pocket cross-talk. The structure of Mdm2 bound by a stapled peptide with a more extended C-terminus does not show any significant ordering of the hinge region to avoid steric clashes [40]. M011 was therefore extended by 3 amino acids at the C-terminus (peptide VIP160), and this resulted in ~1.7 fold improved binding to Mdm4 with no change in affinity for Mdm2 (Table 2). However, this peptide showed a further ~2.5 fold increased binding affinity to Mdm4-L98V, indicating that steric issues in the Trp pocket still prevail for VIP160 bound to Mdm4.

The data show that modification of W^23^ in the context of the stapling chemistry deployed here does not necessarily increase peptide affinity for both Mdm2 and Mdm4. In this respect, alternative macrocyclisation chemistries could be considered [38, 41–44]. Additionally, future iterations of this peptide series should incorporate other non-natural amino acids such as cyclobutyl alanine at the L^26^ position [37], as these could enhance optimal co-engagement of Mdm2 and Mdm4.

## Supporting information

S1 Fig Crystallographic unique complexes of the M011 stapled peptide bound to the N-terminal p53 binding domain of Mdm2-M62A (6–125). The 2Fo-Fc electron density map (blue mesh), contoured at 1.5 σ, clearly demarcates the presence of the whole pepCde (shown using sCck representaCon) bound to Mdm2 (shown using surface representaCon).
(PDF)

S1 File PDB X-ray structure validation summary report. (PDF)
