# Supplementary figures and images for: Structure-activity studies of Mdm2/Mdm4-binding stapled peptides comprising non-natural amino acids
Source: PLoS One. 2017 Dec 11;12(12):e0189379. doi: 10.1371/journal.pone.0189379 (PMC5724825; doi:10.1371/journal.pone.0189379)

Mdm2: Chain A

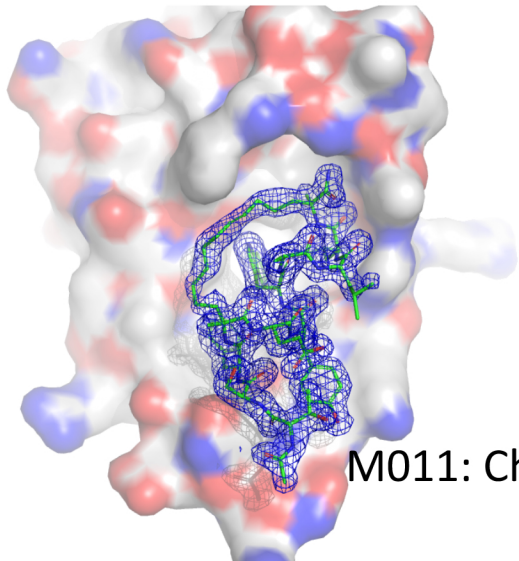

Mdm2: Chain B

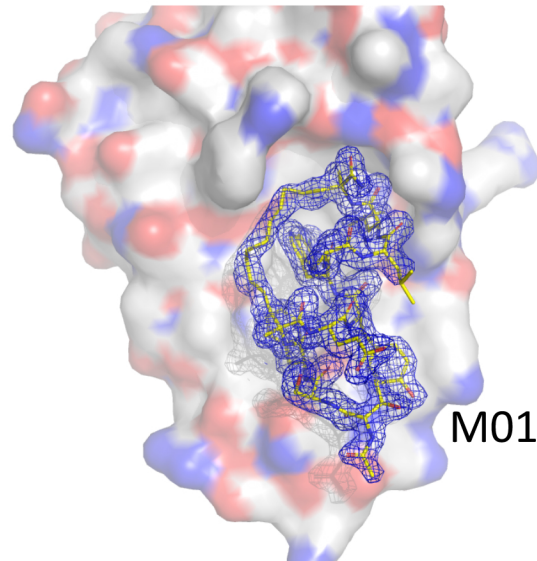

Supplement: S1 Fig — The 2Fo-Fc electron density map (blue mesh), contoured at 1.5 σ, clearly demarcates the presence of the whole pepCde (shown using sCck representaCon) bound to Mdm2 (shown using surface representaCon). (PDF) [file pone.0189379.s001.pdf]
